# Diet as a Preventive of Cholera

**Published:** 1873-10-01

**Authors:** 


DIET AS A PREVENTIVE OF CHOLERA—In a recent number we quoted the senior editor of the *Nashville Journal of Medicine and Surgery*, as saying that "Cholera would not prey upon any one who had no fruits, vegetables, nor animal products in his stomach." As the words *fruits*, *vegetables* and *animal products*, might be interpreted to mean more or less, according to the fancy of the reader, we requested the editor of the Nashville journal to state more definitely what articles of food he would deem safe for use during the prevalence of cholera; and he answers as follows:

"DINNER.

"*First Course.*—Soup-bone soup, with rice boiled in it; salt and pepper to taste.

"*Second Course.*—Roast of beef, broiled spring chickens, loin steak, boiled rice, roasted pullet, cornbread, cold biscuit, iced water.

"*Third Course.*—Coffee, cold biscuit.

"BREAKFAST.

"Mutton chops, fried spring chickens, loin steak, ham and gravy, hot rolls, biscuit, cornbread, baker's bread, rice cakes, with ham gravy, or syrup; coffee, tea, iced water.

"TEA.

"Cold ham, cold boiled chicken, tea or coffee, biscuit, iced water."

In answer to our former rather playful inquiry whether chickens are not *animal products*, Dr. Bowling has the following characteristic assemblage of words: "An animal product is what the creature, animal, produces. God makes a chicken, and a chicken lays an egg. The chicken is God-made—one of His creatures; but the egg is the hen's—a hen's egg, as we all say—an animal product, as I most correctly, most religiously, most decently say."

Conundrums to be answered by our friend Bowling in *Poetry:*

1st. If an egg is an animal product because it is the hen's egg, and a chicken is developed out of the egg, is the chicken the hen's product or the egg's product? In other words, is the chicken the hen's chicken or somebody else's chicken?

2d. If an egg is an animal product because a hen produces it, is not a calf an animal product, because a cow produces it?

Does the hatching of one in the nest, and the other in the uterus, alter the question of product?

3d. If an egg is hen-made, and a chicken God-made, at what stage of the process of incubation does the change take place from the product of the creature, hen, to that of the Creator?

